# Comparison of Grain-Growth Mean-Field Models Regarding Predicted Grain Size Distributions

**DOI:** 10.3390/ma16206761

**Published:** 2023-10-19

**Authors:** Marion Roth, Baptiste Flipon, Nathalie Bozzolo, Marc Bernacki

**Affiliations:** Mines Paris, PSL University, Centre for Material Forming (CEMEF), UMR CNRS, 06904 Sophia Antipolis, France; marion.roth@mines-paristech.fr (M.R.); baptiste.flipon@minesparis.psl.eu (B.F.); nathalie.bozzolo@mines-paristech.fr (N.B.)

**Keywords:** mean-field model, grain growth, grain size distribution, topology, neighborhood description

## Abstract

Mean-field models have the ability to predict the evolution of grain size distribution that occurs through thermomechanical solicitations. This article focuses on a comparison of mean-field models under grain-growth conditions. Different microstructure representations are considered and discussed, especially regarding the consideration of topology in the neighborhood construction. Experimental data obtained with a heat treatment campaign on 316L austenitic stainless steel are used for the identification of material parameters and as a reference for model comparisons. Mean-field models are also applied to both mono- and bimodal initial grain size distributions to investigate the potential benefits of introducing neighborhood topology in microstructure prediction models. This article demonstrates that improvements in the predictions can be obtained in monomodal cases for topological models. In the bimodal test, no comparison with experimental data was performed as no data were available. But relative comparisons between models indicated few differences in the predictions. Although of interest, the consideration of neighborhood topology in grain-growth mean-field models generally results in only small improvements compared to classical mean-field models, especially in terms of implementation complexity.

## 1. Introduction

The phenomenon of grain growth takes place in metallic materials when they are submitted to heat treatment. Considered the only phenomenon that occurs, materials are assumed free of any stored energy (i.e., low dislocation density), and the driving pressure for grain boundary (GB) migration arises from the minimization of the GB surface energy, which leads to the curvature flow problem. At the polycrystalline scale, the GB motion is generally described by $v=M_{GB}\left|P\right|$, where *v* is the velocity norm of the boundary [1], $M_{GB}$ is its mobility, and $P=-\gamma_{GB}\kappa$, where $\gamma_{GB}$ is the GB energy and κ is the trace of the curvature tensor.

For 70 years, models have largely been developed in order to predict microstructure changes under thermomechanical treatments and their impact on macroscopic properties. At the polycrystalline scale, three types of models can be found in the literature: phenomenological, mean-field, and full-field models. Phenomenological approaches are classically based on an experimental database and correspond to the extraction of a mathematical trend from what is observed experimentally. Such models are restricted to the set of thermomechanical conditions and to the material experimentally investigated [2,3]. The last two model types are based on a more generic approach since they use physical equations to predict the evolution of the microstructure. At the mesoscopic scale, full-field models provide access to a complete description of the system, where each individual grain and its topology are taken into account [4,5]. They have the advantage of being able to deal with local heterogeneities. However, their major drawback is that they are very costly in terms of computing time.

Mean-field models, on the other hand, maintain this physically based implementation with a similar set of equations, but the general description of the microstructure is simplified [6,7,8,9,10,11,12]. They also exhibit competitive computation times when compared to full-field models. The original definition of a mean-field model considers the evolution of a microstructure described by mean quantities. The work of Burke and Turnbull [13] provides the simplest version of such models. In this case, the microstructure is defined solely by its mean grain radius $\bar{R}$. The mean grain size (MGS) is commonly determined by the (arithmetic) mean of the equivalent diameter ($\bar{ED}=2\bar{R}$), where $ED_{i}$ is defined in 2D (resp. in 3D) as the diameter of a circle (resp. a sphere) with the same area $A_{i}$ (resp. volume ($V_{i}$)) as the considered grain $G_{i}$:

(1) $$\bar{R}=\frac{\bar{ED}}{2}=\frac{1}{2N}\sum_{i=1}^{N}ED_{i},\;i.e.,\;\;\;\bar{R}=\frac{1}{N}\sum_{i=1}^{N}{\left(\frac{A_{i}}{\pi }\right)}^{1/2}\;\mathrm{in}\;2D,\;\mathrm{and}\;\bar{R}=\frac{1}{N}\sum_{i=1}^{N}{\left(\frac{3V_{i}}{4\pi }\right)}^{1/3}\;\mathrm{in}\;3D,$$

where *N* is the number of grains in the microstructure.

In the Burke and Turnbull (B&T) context, the GB velocity norm is considered proportional to the inverse of $\bar{R}$. The Hillert model [6] introduces the consideration of the grain size distribution (GSD) in mean-field models. The microstructure is sampled in a representative distribution, where each bin is assigned to a given ED value and frequency. In the GSD, a bin is commonly called a grain class, each of them being virtually composed of many grains indicated by the frequency. For every class, one can define a representative grain as having the characteristics of the associated grain class. The Hillert formalism represents the microstructure as a grain embedded in a Homogeneous Equivalent Medium (HEM) that is characterized by the MGS of the distribution. The size evolution of a considered class is deduced from the difference in the curvature radius between the current class and that of the HEM. Later on, statistical models, such as the one developed by Abbruzzese et al. [7,14], modified the definition of the HEM. Rather than only being associated with a mean grain radius, the HEM is replaced with contact surfaces defined for all grain classes in the microstructure. Each grain class is surrounded by a statistical medium (SM) composed of all the grain classes in the microstructure. The contact surface between a grain class and its surrounding neighbors is defined by a perimeter intersection probability. One of the latest topological mean-field models, developed by Maire et al. [12], combines several formalisms in its neighborhood construction. This hybrid model uses both the statistical approach initiated by Abbruzzese et al. and a deterministic number of neighbors ruled by the imposed bijectivity of neighborhood assignment. To the best of the authors’ knowledge, the significance of these different views concerning the neighborhood description has not been discussed in the state of the art for GG modeling. The main purpose of this work is to compare mean-field models of different microstructure descriptions to determine whether semi-topological approaches can better describe the evolution of GSD in the context of grain growth. A further aim of this article is to dissect the various nuances of pre-existing models in the state of the art, which is not as simple a task as it might seem, and thus to guide the reader based on their expectations of the desired model.

This article is constructed as follows. First, mean-field GG models are introduced. A detailed description of the microstructure formalism in Hillert, Abbruzzese et al., and Maire et al. models is presented. Then, optimized data parameters for the use of these models are determined and discussed. The last section is dedicated to comparing the distribution of the Maire et al. model with the mean-field models of Hillert and Abbruzzese et al., as well as experimental data.

## 2. Mean-Field Models

This section recalls the main equations and details the microstructure description of the previously introduced models for the GG phenomenon.

### 2.1. Burke and Turnbull Model

The B&T model [13] illustrates the original meaning of mean-field models, as the microstructure description is reduced to mean geometric considerations. Indeed, the rate of GG ($d\bar{R}/dt$) is assumed to be proportional to $1/\bar{R}$ [1] as follows:

(2) $$d\bar{R}=M_{GB}P_{c}dt,\;\mathrm{with}\;P_{c}=\frac{\alpha \left(d-1\right)\gamma_{GB}}{\bar{R}},$$

where α is a constant, $\gamma_{GB}$ is the GB energy, $M_{GB}$ is the GB mobility, and *d* is the space dimension. In the B&T analysis, $\gamma_{GB}$ and $M_{GB}$ are assumed to be constant in time and space.

A parabolic expression can then be derived from Equation (2) and represents the time dependence of ${\bar{R}}^{2}-{{\bar{R}}_{0}}^{2}$:

(3) $${\bar{R}}^{2}-{{\bar{R}}_{0}}^{2}=2\left(d-1\right)\alpha M_{GB}\gamma_{GB}t,$$

where ${\bar{R}}_{0}$ is the initial mean grain radius. An extension of this law is classically preferred in the literature:

(4) $${\bar{R}}^{2}-{{\bar{R}}_{0}}^{2}=\tilde{\alpha }M_{GB}\gamma_{GB}t^{n},$$

where *n* and $\tilde{\alpha }$ are two constants to be fitted using experimental data.

### 2.2. Hillert Model

**Figure 1:**
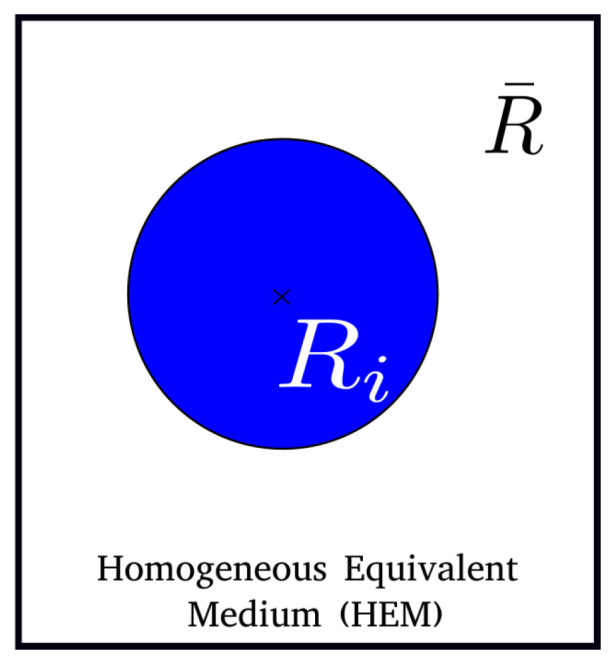
Schematic representation of the microstructure in the Hillert model.

The GG mean-field model proposed by Hillert [6] relies on considering each grain class within a common HEM. The microstructure description is illustrated in Figure 1, where the representative grain of a given grain class $R_{i}$ is surrounded by an HEM defined by the mean grain radius of the microstructure $\bar{R}$. Compared to the B&T model, the driving pressure term is modified using a more local approach. $P_{c}$, for class *i*, is defined as a function of the curvature radius of grain class *i*:

(5) $$dR_{i}=\frac{\left(d-1\right)}{2}M_{GB}\gamma_{GB}\left(\frac{1}{\bar{R}}-\frac{1}{R_{i}}\right)dt.$$

Thus, grain *i* will shrink when $\bar{R}>R_{i}$, it will grow when $\bar{R}<R_{i}$, and it will be stable when $\bar{R}=R_{i}$.

### 2.3. Abbruzzese et al. Model

**Figure 2:**
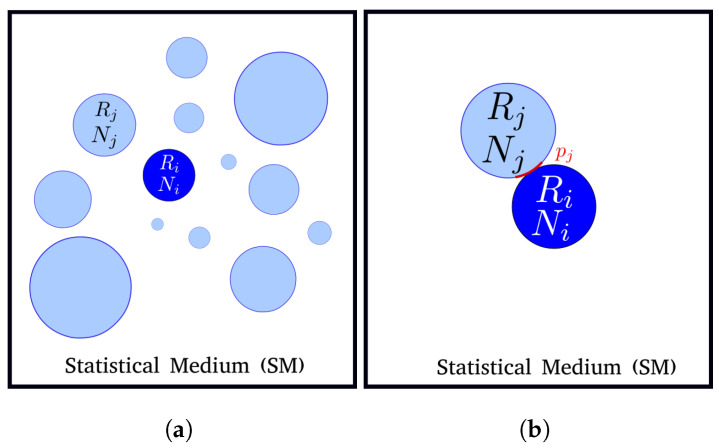
Statistical neighborhood construction of the 2D GG model of Abbruzzese et al.: (**a**) the statistical medium, and (**b**) the concept of the contact probability $p_{j}$.

In the statistical model proposed by Abbruzzese et al. [7,14] in 1992, the authors suggested an evolution of the Hillert model by introducing a statistically constructed medium composed of all grain classes in the microstructure, as illustrated in Figure 2a. The contribution of all neighbors is weighted by a statistical coefficient: the contact probability.

In 2D, considering grain class *i*, the contact between *i* and its neighbor grain classes is defined by a fraction of the neighbor class perimeter. The probability of grain class *j* belonging to the SM is dependent only on its own size (grain radius—$R_{j}$) and will be the same for all grain classes *i* considered. This represents the likelihood or probability of a grain from the *j*th class in the microstructure intersecting the representative grain *i*, as schematized in Figure 2b. The contact probability $p_{j}$ is then determined by the ratio of the perimeter of the *j*th grain to the sum of all grain perimeters in the microstructure. Along with the grain size, the frequency of occurrence of each grain class is also taken into account. The expression for $p_{j}$ is as follows:

(6) $$p_{j}=\frac{N_{j}R_{j}}{\sum_{k=1}^{n}N_{k}R_{k}},$$

where *n* is the total number of grain classes in the microstructure, and ∀k∈〚1,n〛, $N_{k}$ is the number of grains belonging to grain class *k*.

With such an explicit description of the surroundings, the GB migration can be achieved locally between class *i* and each of its neighbors. In the context of 2D GG (d=2), the difference in the driving pressure between grain class *i* and its neighbor *j* can be derived from the Hillert equation (Equation (5)) and is given by:

(7) $$dR_{\left(i,j\right)}=\frac{1}{2}M_{GB}\gamma_{GB}\left(\frac{1}{R_{j}}-\frac{1}{R_{i}}\right)dt.$$

The global variation in the grain size undergone by grain class *i* is the sum of the local variations with each *j*th neighbor. The local GB migration can be generalized to all *j*th neighbors of grain class *i* using the contact probability $p_{j}$. The total rate of evolution with respect to time for the radius of the representative grain *i* can then be written as:

(8) $$dR_{i}=\sum_{j=1}^{n}p_{j}dR_{\left(i,j\right)}=\frac{1}{2}M_{GB}\gamma_{GB}\left(\sum_{j=1}^{n}\frac{p_{j}}{R_{j}}-\frac{1}{R_{i}}\right)dt.$$

### 2.4. Maire et al. Model

Maire et al. [12] proposed a 3D mean-field model to simulate the microstructure evolution under thermomechanical solicitations. Physical mechanisms, such as GG, discontinuous dynamic recrystallization, and post-dynamic recrystallization, can be modeled. The model is based on previous works by Bernard et al. [10] and Beltran et al. [15] who developed a mean-field model with two HEMs; one medium was associated with non-recrystallized grains and the other was associated with recrystallized grains, each of which was characterized by their MGS. The work of Maire et al. focused on the introduction of neighborhood topology in the previously defined Bernard–Beltran formalism. A specific neighborhood was proposed for each grain class in the microstructure. Each class *i* was characterized, in the context of GG, using two main properties: the grain radius $R_{i}$ and the number of grains belonging to this class $N_{i}$. The microstructure and its evolution are described below. The following equation links the radius variation $dR_{\left(i,j\right)}$ between two representative grains to the volume variation $dV_{\left(i,j\right)}$ associated with the GB migration:

(9) $$dV_{\left(i,j\right)}=dR_{\left(i,j\right)}\times S_{c(i,j)}=dR_{\left(i,j\right)}\times p_{\left(i,j\right)}\times S_{R_{i}},$$

(10) $$\mathrm{with}\;dR_{\left(i,j\right)}=M_{GB}\gamma_{GB}\left(\frac{1}{R_{j}}-\frac{1}{R_{i}}\right)dt,\;\mathrm{as}\;d=3\;(\mathrm{see}\;\mathrm{Equation}\;(5)),$$

And, $S_{c(i,j)}$ represents the contact surface between class *i* and *j*, which is the product of $p_{\left(i,j\right)}$, the contact probability, and $S_{R_{i}}$, the remaining surface of grain class *i*:

(11) $$S_{c(i,j)}=p_{\left(i,j\right)}\times S_{R_{i}}.$$

**Figure 3:**
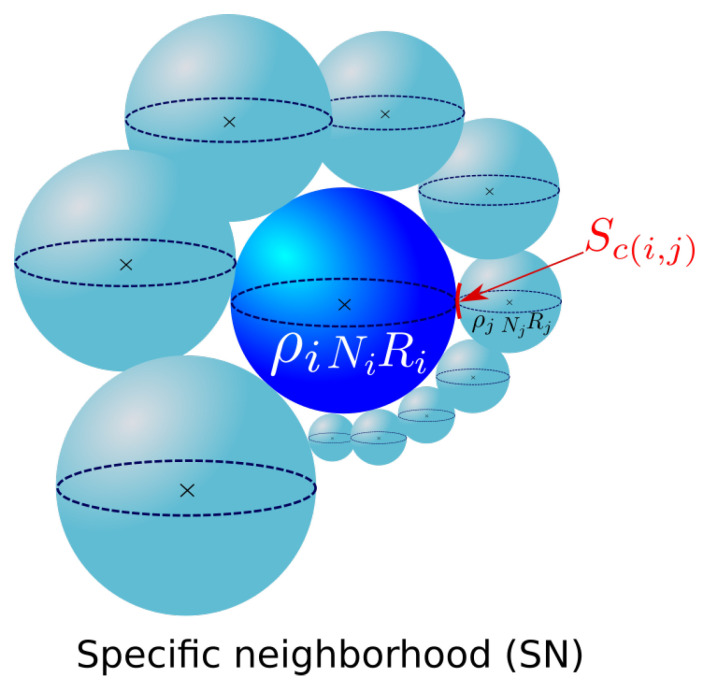
Microstructure description in the Maire et al. model.

Figure 3 illustrates the microstructure description considered in the Maire et al. model.

The model considers a specific neighborhood (SN) built for each grain class based on two main criteria. First, bijectivity is ensured between two neighbor classes. For two considered neighbor grain classes *i* and *j*, the contact probability of grain class *i* with grain class *j* is computed as $p_{\left(i,j\right)}$, and it is automatically associated with grain class *j* as the contact probability $p_{\left(j,i\right)}=p_{\left(i,j\right)}$. The second criterion imposed by this model that is intimately linked to the first one is volume conservation, which is defined in more detail below.

At each time increment of a grain-growth simulation, the surface of the spherical representative grain of a class is filled with a neighborhood. Initially, each grain class has a remaining (available) surface equal to the surface of a sphere of radius $R_{i}\left(t=0\right)$: $S_{R_{i}}=4\pi R_{i}^{2}$. During the SN construction, the remaining available surface will decrease as neighbors are assigned until the SN of the considered grain class is completely filled (i.e., no more available surface).

The filling of the SN is arbitrarily selected to be carried out in ascending order of the GSD. By considering *i* as the current class, a first neighbor class i+1 is attributed to class *i*. The corresponding contact surface $S_{c\left(i,i+1\right)}$ is applied between the two considered grain classes, and $S_{R_{i}}$ is updated as follows:

(12) $$S_{R_{i}}=4\pi R_{i}^{2}-S_{c\left(i,i+1\right)},$$

The $S_{c\left(i,i+1\right)}$ computation is described in more detail below.

**Figure 4:**
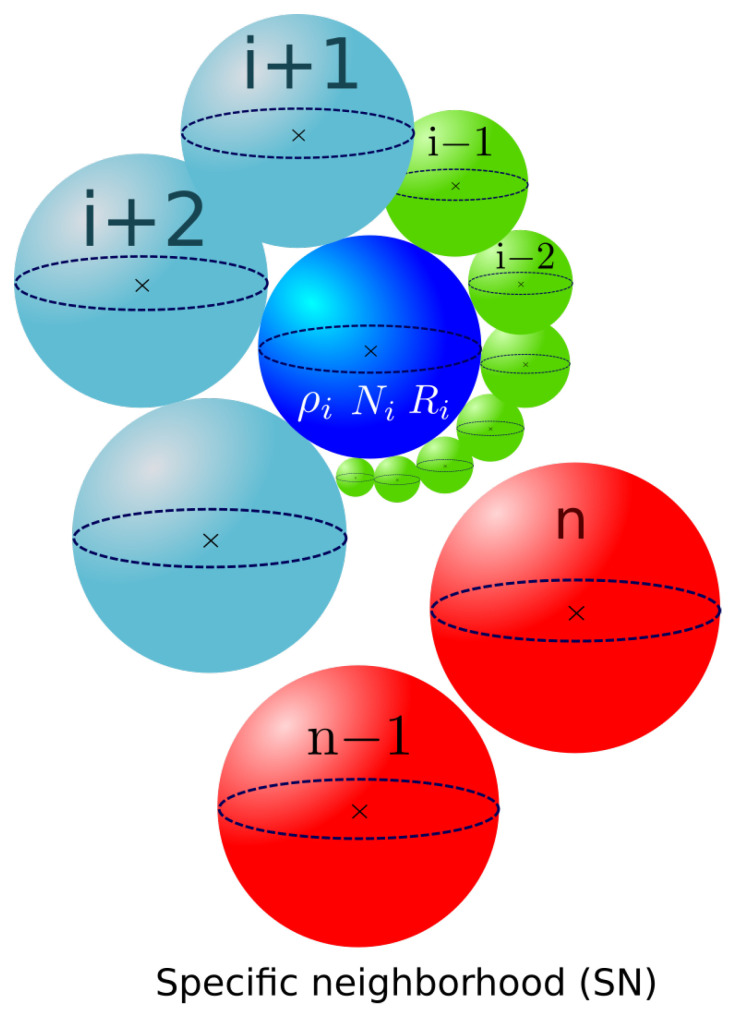
Specific neighborhood construction in the Maire et al. model for class *i*.

The next neighbors, j=i+2,i+3,…, for class *i*, are then selected in ascending order of the initial GSD, and a contact surface is computed once more. This applies to all microstructure grain classes with incomplete neighborhoods. Once the *i*th grain class neighborhood is filled, the following class, denoted here as i+1, becomes the current class, and so on until the entire grain distribution is scanned. As the current class progresses in the GSD, a part of its neighborhood is already filled up due to bijectivity with classes of smaller radii. In Figure 4, the current class *i* considered is positioned in the middle of the GSD. The green classes represent those attributed to grain class *i* by bijectivity when i−1,i−2,… were the current grain classes. The blue classes are all the remaining classes of the distribution with no complete neighborhoods. These classes are added to the grain *i* neighborhood when it is the current class. Finally, the red grain classes do not belong to the grain class *i* neighborhood, as their neighborhoods are already complete due to bijectivity with smaller radius grain classes.

As mentioned in the introduction, a bijective criterion is applied here. The remaining surface of grain class i+1, $S_{R_{i+1}}$, has already been updated when i+1 was attributed as the neighbor to grain class k,withk≤i. This means that as the current class progresses in the distribution, a part of the neighborhood has already been filled due to bijectivity with grains having a smaller radius than the current class. After the update, the remaining surface of grain class *j* progressively decreases. A test is conducted to check if $S_{R_{j}}>0$; if true, grain class *j* can take new neighbors; if not, the grain class *j* neighborhood is complete. Class *j* is, therefore, no longer available as a neighbor and will not take part in the construction of subsequent grain classes (class *j* will be represented as the red grain classes in Figure 4 for all other classes).

Of course, it is important to highlight here that the order used to build the neighborhood of each class can have an impact on the overall outcome of the GG model. For example, the neighborhood determination will be different if the distribution is processed in decreasing order of GSD. This effect is discussed below.

Equation (9) introduces the contact probability $p_{\left(i,j\right)}$ between two grain classes in the microstructure. While the concept of contact probability by Abbruzzese et al. has been conserved in equation form, the spatial dimension of contact has been elevated to a volumic consideration, as expressed in the following equation:

(13) $$p_{\left(i,j\right)}=\frac{N_{j}R_{j}^{3}}{\sum_{k=1}^{n_{i}}N_{k}R_{k}^{3}},$$

where $n_{i}$ is the number of grain classes with an incomplete neighborhood when the *i*th grain class neighborhood is constructed.

As previously presented, this model is described in 3D, so the quantities exchanged during GB migration are volumetric. The volume variation experienced by a grain is the sum of all signed volume variations with respect to its neighbors, as defined by Equation (9):

(14) $$\Delta V_{i}=\sum_{j=1}^{\eta_{i}}dV_{\left(i,j\right)}$$

where $\eta_{i}$ is the number of neighbors of grain class *i*. As $dV_{\left(j,i\right)}=-dV_{\left(i,j\right)}$ owing to the imposed bijectivity, the volume conservation of the global system is ensured.

## 3. Input Data for Mean-Field Modeling

### 3.1. Material-Dependent Model Parameters Acquisition

Experimental data and material parameter identification are necessary for calibrating a mean-field model for a given material and a temperature range. This section details the material and experimental data used in determining the model parameters.

#### Experimental Data

A single-phase austenitic stainless steel (316L) was selected for this study. The identification procedure of the reduced mobility product ($M_{GB}\gamma_{GB}$) presented in this work required a minimal campaign of nine thermal treatments [16]. Fifteen are provided here and their conditions are detailed in Table 1. Longer annealing times from 3 to 5 h were conducted to validate the identification procedure for long durations. These heat treatments were performed using a Carbolite furnace. A thermocouple was placed in the furnace near the samples to control and record the temperature evolution. It is, of course, important to clarify that the proposed experimental methodology and the analysis of the results using the discussed mean-field models in the following sections are fully applicable to other austenitic steels and, more generally, to any polycrystalline material subject to an isotropic grain-growth mechanism.

**Figure 5:**
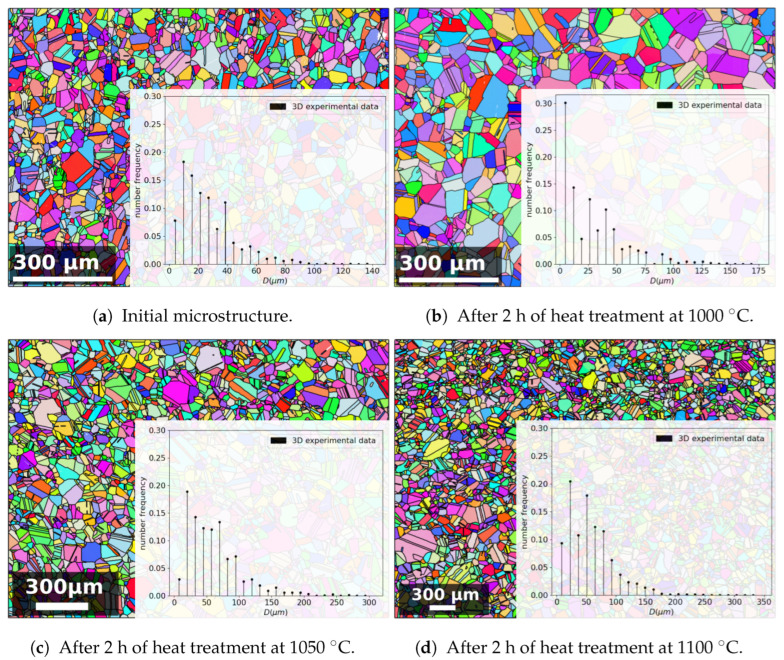
In the background: IPF Z maps of 316L microstructures of (**a**) the as-received material, (**b**) after 2 h at 1000 °C, (**c**) after 2 h at 1050 °C, and (**d**) after 2 h at 1100 °C. The black lines denote the grain boundaries. In the foreground: the corresponding 3D GSD after an inverse Saltykov transformation.

These samples were prepared for electron back-scattered diffraction (EBSD) analyses by cutting and selecting a centered observation area to avoid the effects of surface oxides on the analyses. The classical first steps involved polishing the stainless steel using abrasive SiC paper, followed by polishing with a 3 μm diamond suspension, and finally, electropolishing for 25 s at 10 V with a solution consisting of 10% perchloric acid in ethanol. The EBSD analyses were performed with a Carl Zeiss Supra 40 field emission gun scanning electron microscope (FEGSEM) coupled with a Bruker Quantax EBSD detector and Esprit 2.3 software. A voltage of 20 kV and a 120 μm aperture were used. Post-processing of the EBSD data was performed using the MTEX Matlab toolbox [17]. The step size and cartography dimensions were chosen to obtain a representative number of grains in the observation area. Table 2 presents these parameters, as well as the number of grains observed in each cartography. This count was calculated without taking into account the twin boundaries and using a misorientation of 15° to define a grain boundary. The minimal size for a grain to be considered an entity was greater than 5 pixels. The processes of entity removal under the set threshold and grain boundary smoothing were applied, considering only indexed pixels. These data also provided access to the 2D GSDs that were transformed into 3D using the Saltykov algorithm (detailed in the following section). These distributions were used not only as the initial input for the model but also as experimental data for comparison with the simulation results in Section 4. Figure 5 displays the IPF Z maps of some of the post-treated experimental results, as well as the related histograms depicting the frequency of occurrence.

### 3.2. Use of Saltykov Algorithm to Obtain a 3D GSD

**Figure 6:**
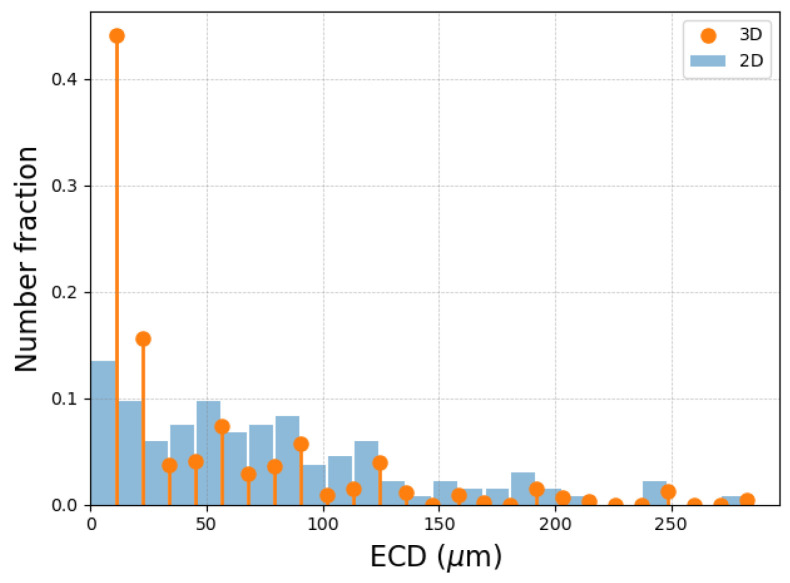
Inverse Saltykov method illustrated on the 2D GSD of a sample heat-treated at 1050 °C for 5 h. The blue histogram corresponds to the 2D GSD and the orange discrete distribution corresponds to the 3D results obtained through the inverse Saltykov algorithm.

The experimentally acquired EBSD data represented 2D slices of 3D polycrystals. To be consistent with the mean-field simulation results and to enable comparisons, a 2D-to-3D conversion was performed. To this end, the Saltykov method [18] was applied to the experimental GSDs. The method was initially developed for extracting 2D section data from 3D granulometry data of spherical particles. The inverse Saltykov method allows for the transformation of GSD data from a 2D histogram distribution into a 3D discrete distribution [18]. The method relies on several assumptions, which are compatible with the topology of the initial 316L microstructure under consideration. The Saltykov method has indeed been proven to be efficient on a similar equiaxed polycrystal [19]. No assumption is required regarding the shape of the input distribution, so multi-modal distributions can also be submitted to such a method [20]. The methodology ensures that the average of an infinite number of 2D cuts of a polycrystal respecting the obtained 3D discrete distribution will converge toward the imposed 2D histogram distribution. However, the quality of the methodology is, of course, also linked to the statistical representativity of the input 2D GSD. This procedure is illustrated in Figure 5, showing the evolution of the 3D distribution after the 2 h thermal treatments at the various temperatures summarized in Table 2. Moreover, Figure 6 illustrates the difference between the 2D histogram distribution and the 3D discrete distribution obtained through the inverse Saltykov transformation.

Moreover, for one particular microstructure (*T* = 1050 °C for *t* = 5 h), Figure 6 illustrates a comparison between the 2D histogram distribution (in blue) and the 3D discrete distribution (in orange) obtained through the inverse Saltykov transformation. The 3D discrete diameters are positioned at the maxima of the 2D bins.

#### 3.2.1. GB Mobility Parameter Identification

##### A First Approximation Using the Classical B&T Law

The first step in the GB mobility identification procedure is to find an initial approximation for the reduced mobility $\left(M_{GB}\gamma_{GB}\right)$ in order to run a first mean-field computation. To this end, the historical form of the Burke and Turnbull law [1,13] (Equation (4) with $\tilde{\alpha }=1/2$ and n=1) is used to obtain a first approximated value of the reduced mobility for each considered temperature (see Table 1).

**Figure 7:**
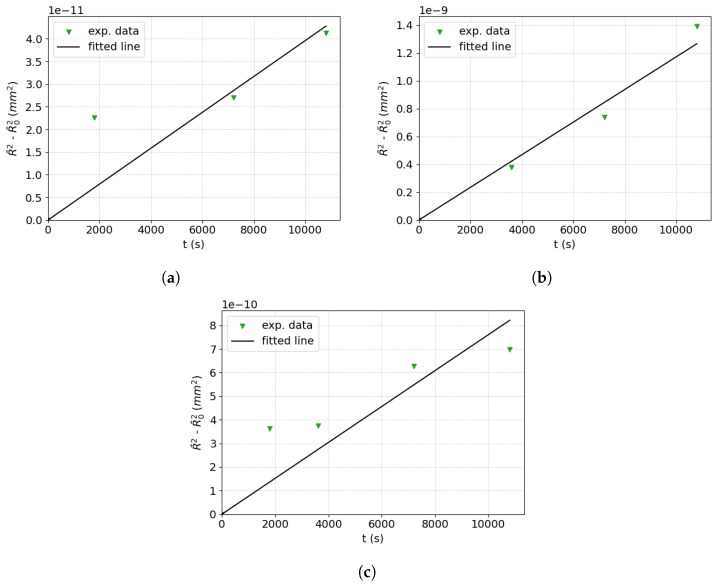
Use of the Burke and Turnbull law to obtain the first value of ${\left(M_{GB}\gamma_{GB}\right)}_{ini}$ for 316L at (**a**) 1000 °C, (**b**) 1050 °C, and (**c**) 1100 °C.

For each heat treatment temperature, the B&T law is plotted in order to obtain a linear dependence between ${\bar{R}}^{2}-{{\bar{R}}_{0}}^{2}$ and the time *t*. Figure 7 illustrates the methodology on sets of experimental points for the three studied temperatures, where the best linear fit directly provides a first rough value for $\left(M_{GB}\gamma_{GB}\right)$ called ${\left(M_{GB}\gamma_{GB}\right)}_{ini}$. Following this strategy, Table 3 summarizes the initial values obtained for the Maire et al. model in ascending order of the initial GSD for the investigated temperatures for 316L.

##### Refined Identification

**Figure 8:**
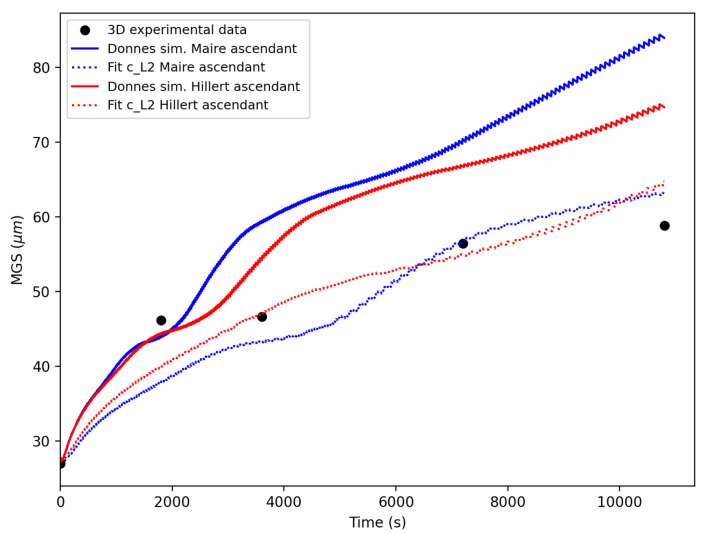
Curve fitting of the simulation points (obtained with ${\left(M_{GB}\gamma_{GB}\right)}_{ini}$) with respect to the experimental points by minimizing *L${}^{2}$* error for the Maire et al. and Hillert models at 1050 °C for 316L.

The values ${\left(M_{GB}\gamma_{GB}\right)}_{ini}$ are then used in the Hillert and Maire et al. models for comparison with the experimental points (solid blue and red lines, respectively, in Figure 8). These first simulation results are then used to perform an optimization of the reduced mobility by calculating the $L^{2}$ error on several points between the simulation and experimental results. A translation coefficient $c_{fit}$ is defined using the least-square method to shift the simulated curve in order to improve the correlation with the experimental data so that:

(15) $${\left(M_{GB}\gamma_{GB}\right)}_{fit}=c_{fit}\times {\left(M_{GB}\gamma_{GB}\right)}_{ini}.$$

The cost function of the least-square method is as follows:

(16) $$F\left(c\right)=\sum_{i=1}^{n}f_{i}^{2}\left(c_{i}\right),$$

where *n* is the number of experimental points, $c_{i}=\frac{t_{sim_{i}}}{t_{exp_{i}}}$ is the translation coefficient between the interpolated curve of the simulation data and the experimental points, and $f_{i}\left(c_{i}\right)$ represents the $L^{2}$ errors between the simulated and experimental points for each value of $c_{i}$:

(17) $$f_{i}\left(c_{i}\right)=L_{i}^{2}\left(c_{i}\right)=100\times \sqrt{\frac{\sum_{k=1}^{n}{\left(\frac{t_{sim_{k}}}{c_{i}}-t_{exp_{k}}\right)}^{2}}{\sum_{k=1}^{n}t_{exp_{k}}^{2}}}\;\mathrm{with}\;\forall i\in 〚1,n〛.$$

##### Model-Dependence of Reduced Mobility

Since the intrinsic GB mobility of a material is hard to quantify experimentally, a common approach is to model the evolution of the GB migration (for instance, using the $v=M_{GB}P$ equation) and consider the mobility $M_{GB}$ as a material-dependent model parameter. Depending on the definition used in the pressure term *P*, the mobility will also be a model-dependent parameter.

The solid lines in Figure 8 illustrate the outcomes of the Hillert and Maire et al. models when subjected to an identical mobility value. The corresponding colored, dashed lines represent the outcomes after identifying the mobility for each model. The reduced mobility values for a temperature of 1100 °C are presented in Table 4. The GB migration equations in these models (c.f. Equations (5), (7), and (10)) are rather similar, accounting for the identified reduced mobility being of the same order of magnitude.

## 4. Results and Discussion

This section is dedicated to the optimization of the numerical parameters and to a comparison of the results obtained using the different mean-field models for 316L austenitic stainless steel in the context of GG. The various heat treatment conditions (temperature, duration) in Table 1 are simulated and compared with the experimental GSDs.

### 4.1. Numerical Parameters

As described in Section 2, the three mean-field models (Hillert, Abbruzzese et al., and Maire et al.) are based on specific media or neighborhoods. These neighborhoods can influence several modeling parameters, and a thorough study is required to either optimize their values or evaluate their impact on model predictions.

#### 4.1.1. Convergence Study Concerning the Number of Grain Classes Introduced in the Model

An important parameter common to these models is the initial number of grain classes introduced at the start of a simulation. Statistical representativity is determined by two criteria: the minimum number of grain classes necessary for a GSD to accurately represent an experimental microstructure and the necessity to represent the different grain populations existing in the material (detection of mono- or multimodal distributions). The convergence study performed here focuses on the first criterion. In terms of GG, the number of grain classes decreases over time due to capillarity effects; therefore, the representativity of the microstructure is affected. The Maire et al. neighborhood construction relies on good statistical representativity, as it widens the choice of classes in the selection of neighbors. A convergence study was performed on this parameter to determine the minimal initial number of grain classes necessary to obtain a reproducible final GSD. Thermal treatment of one hour at 1100 °C was used for this discussion, and the GSD results were compared to a reference simulation for each model. More precisely, seven simulations from 25 to 2000 initial grain classes were run and compared to a reference simulation where 5000 grain classes were considered. This reference was considered representative of the experimental values. Indeed, the number of grains in the sample area examined in the EBSD analysis varied from 3500 to 130 grains, with an average of around 1400 grains for all the studied conditions presented in Table 2. The use of 5000 initial grains classes perfectly covered the 980 grains observed experimentally. Also, a final number of 1300 classes was obtained through this simulation after 5 h.

**Figure 9:**
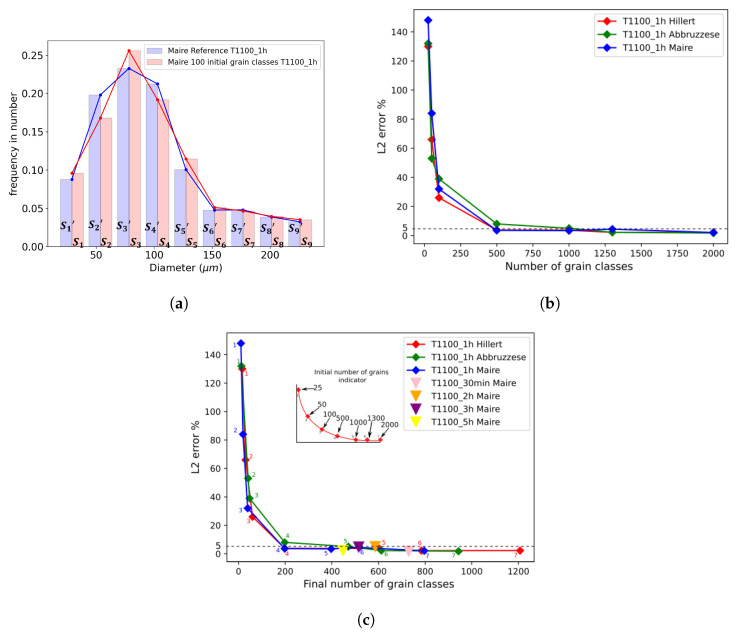
(**a**) Histogram of the $L^{2}$ (1 h) error method for analyzing the convergence of simulations at 1100 °C, (**b**) convergence study of the initial number of grain classes by computing the $L^{2}$ (1 h) error at 1100 °C for the Hillert, Abbruzzese et al., and Maire et al. models on 316L, and (**c**) the same convergence study computing the $L^{2}$ (1 h) error for the three models using the final number of grain classes as the *x*-axis.

To determine the value at which convergence was reached, Equation (18) provides the relative error at time *t* between the reference case and the tested GSDs:

(18) $$L^{2}\left(t\right)=100\times \sqrt{\frac{\sum_{i=1}^{n_{\mathrm{bins}}}{\left(S_{i}-S_{i}^{\prime }\right)}^{2}}{\sum_{i=1}^{n_{\mathrm{bins}}}{\left(S_{i}^{\prime }\right)}^{2}}},$$

where $n_{\mathrm{bins}}$ is the number of bins in the histograms used for comparison. This number ($n_{\mathrm{bins}}$) was introduced to simplify the visual representation of the histograms and facilitate a meaningful comparison of the simulations. A reduced number of histogram bins was then selected relative to the total number of grain classes. Typically, for the histogram representations presented in this paper, with the exception of Figure 9a, $n_{\mathrm{bins}}$ was set to 25. The corresponding bin width was computed from this number. This allowed for the recreation of a unique ECD vector of 25 visually distinct grain classes, each an equal distance from the other, to accurately compare the GSDs.

In Figure 9a, a total of nine bins were used to simplify the visual explanation for the $L^{2}$ comparison. In this figure, $S_{i}$ (resp. $S_{i}^{\prime }$) corresponds to the *i*th bin grain class area of the GSD at *t* = 1 h for the Maire et al. model (resp. the reference GSD of the model).

Figure 9b plots the $L^{2}$ (1 h) evolution for the different initial numbers of grain classes in each model at 1100 °C. From 25 to 500 initial grain classes, the $L^{2}$ (1 h) error decreased drastically from above 100% to 3%. A total of 25 initial grain classes resulted in an $L^{2}$ (1 h) error greater than 100% for all models, thereby reducing the degradation of the statistical representativity. The convergence threshold was set at 5%, considering that convergence was reached below that error. From 500 to 2000 initial grain classes, the simulations therefore converged, considering the threshold. For the comparisons performed in Section 4, an initial number of 1000 grain classes was chosen to assure consistency and computational time efficiency. This value was also in perfect agreement with the experimental initial number of grains of 980, as presented in Table 2. The statistical representativity also relied on a second parameter, which corresponded to the number of grain classes present at the end of a numerical computation.

Figure 9c illustrates the same convergence study as that depicted in Figure 9b but with the *x*-axis representing the number of final grain classes at the end of each simulation. This shows that a minimum number of 200 remaining grain classes was necessary to remain below the threshold of 5% of the $L^{2}$ (1 h) error. As the model operated based on grain classes, it can be considered that 200 classes can effectively describe the range of the experimental number of grains from 130 to 3500 observed in the EBSD maps under different conditions. This figure also shows that for an identical initial number of grain classes, the Hillert model retained a higher number of remaining grain classes, implying better statistical representativeness.

For the subsequent GSD comparisons, the statistical representativity of heat treatment from 2 to 5 h at 1100 °C with an initial number of 1000 grain classes was also verified using the Maire et al. model, as illustrated in Figure 9c with colored triangle icons. Indeed, one can see that for the three thermal treatments, the previously defined threshold for representativity was well maintained, as the $L^{2}\left(t\right)$ error (in comparison to the reference case at 5000 grains classes), remained below 5%, and the remaining number of classes after the annealing time was above 200.

#### 4.1.2. Different Spatial Dimensions Considered to Define the Contact Probability

##### Description of the Spatial Dimensions

In the original work of Abbruzzese et al. [7], the 2D contact probability was computed based on the perimeter of the neighbor class (c.f. Section 2.3). This formalism was extended to 3D by considering sphere surfaces instead of disk perimeters in Di Schino et al. [21]. The contact probability construction suggested by Maire et al. is a generalization of this formalism. This section focuses on determining whether the contact probability has a positive effect on refining the description of the Maire et al. model-simulated GSDs. Inspired by the work of Abbruzzesse et al. in Equation (6), four types of contact probabilities can be derived, ranging from a numerical probability to a volumetric probability:

(19) $$p_{\left(i,j\right)}^{m}=\frac{N_{j}R_{j}^{m}}{\sum_{k=1}^{n_{i}}N_{k}R_{k}^{m}}\;\mathrm{with}\;m\in 〚0,3〛,$$

where $N_{j}$ is the number of grains belonging to grain class *j*, and $n_{i}$ is the number of grain classes with an incomplete neighborhood when $p_{\left(i,j\right)}^{m}$ is computed for grain class *i*.

**Figure 10:**
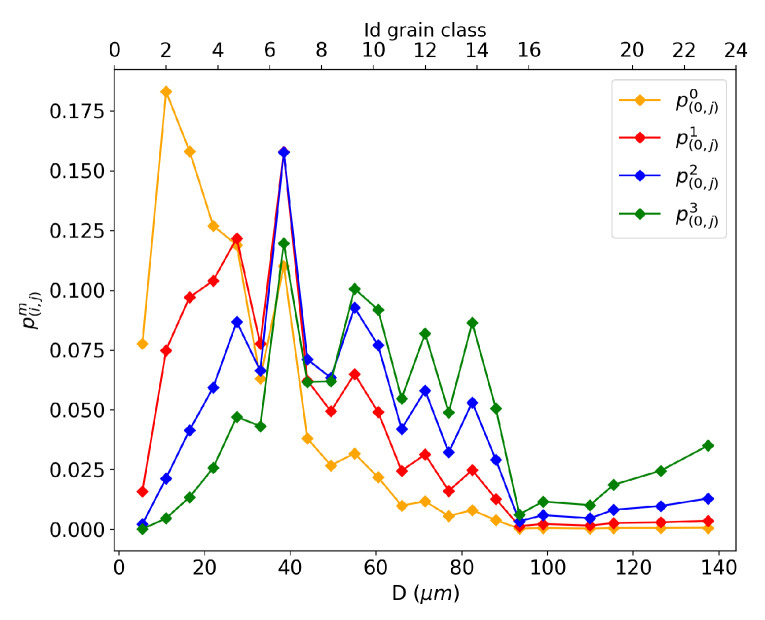
Description of the different contact probabilities $p_{\left(i,j\right)}^{m}$ with neighbor classes of the first class (i=0) in the microstructure at *t* = 0 s.

The four contact probability definitions, as described by Equation (19), pertain to the neighborhood of the first grain class in the microstructure at the initial time *t* = 0 s, as plotted in Figure 10. These probabilities are used in the context of the Maire et al. model, as detailed in Section 2.4, by modifying Equation (13). In Equation (19) with m=0, the contact probability $p_{\left(i,j\right)}^{0}$ is computed solely in terms of numbers, with no influence from the grain size. In Figure 10, the orange curve shows that the majority of the weight is assigned to smaller neighboring grains, accounting only for their frequency of occurrence in the microstructure. The $p_{\left(i,j\right)}^{1}$ formulation uses the perimeter of the grain class to describe the contact probability. This formulation assigns the highest probability values to the first one-third of grain classes, indicating that small grain classes are still more represented in the neighborhood of the first class in this description. The $p_{\left(i,j\right)}^{2}$ formulation considers the surface of the neighboring grain class *j* to build the contact probability. A shift toward the middle-sized grains is observed, leading to larger grains taking part in the neighborhood compared to the $p_{\left(i,j\right)}^{0}$ and $p_{\left(i,j\right)}^{1}$ definitions. Finally, the description $p_{\left(i,j\right)}^{3}$ establishes a neighborhood based on the volume of the neighboring grain classes, giving more weight to larger grain classes, as highlighted by the green curve in Figure 10. Looking solely at the neighborhood construction, none of these representations seems more justified than the others. Depending on the selected representation, certain groups of grains are emphasized, as previously explained.

##### Impact on the Distribution Results

**Figure 11:**
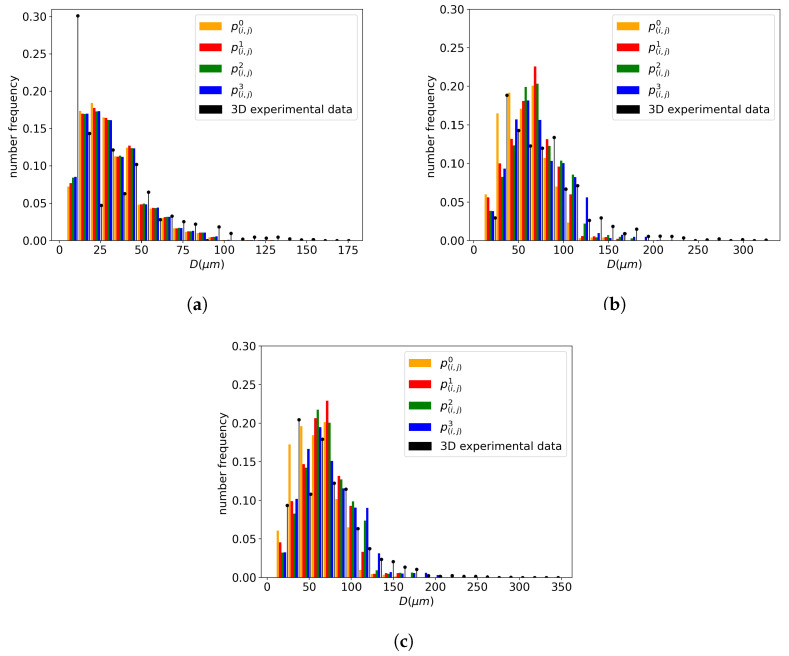
Comparison of the impact on the distribution of the $p_{\left(i,j\right)}^{m}$ with m∈〚0,3〛 for an annealing time of 2 h at different temperatures for 316L: (**a**) 1000 °C. (**b**) 1050 °C. (**c**) 1100 °C.

To select one of the above contact probability definitions for the study, the grain size distribution results are compared using a GG test case. For the three investigated temperatures (1000, 1050, and 1100 °C) an annealing of two hours was simulated using the four contact probabilities. The GSD results were then compared to the data converted into a 3D GSD, as shown in Figure 11, using the Saltykov method described in Section 3.2. The experimental data are represented by a discrete black histogram. At all three temperatures, $p_{\left(i,j\right)}^{3}$ seems to provide a better fit of the tail of the distribution. This volume-based description, derived from Equation (19) with m=3, accentuates the topological effect of the neighborhood construction by assigning more weight to bigger grains. If a homogeneous microstructure is composed solely of small grains, the contact probability description will yield similar contact probabilities for these grains with a comparable volume. However, if a heterogeneous microstructure containing both large and small grains is considered, the volumetric probability introduces a topological aspect by assigning more weight to the larger grains. Indeed, if the larger grains have a better representation in the neighborhood of a grain class, then the dV exchange achieved with the GB migration Equation (9) for these grain classes is increased. Therefore, they statistically have a higher likelihood of growing and being represented in the distribution in subsequent time steps.

#### 4.1.3. Impact of the Selection Order of Grain Classes on the Neighborhood Construction

The complexity of the neighborhood construction proposed by Maire et al. is influenced by the selection order of the grain classes in the GSD. As mentioned in Section 2.4, in the original work of Maire et al. [12], ascending order is arbitrarily selected for the GSD. For the Hillert and Abbruzzese et al. models, the MGS evolution with respect to time remains unchanged, regardless of the order in which the grain classes are selected, as no topology is involved in the computation of their surrounding media.

To study the effect of the selection order of the grain classes on the results of the neighborhood construction, two other types of selection orders were considered: the GSD was either selected by decreasing grain sizes or randomly.

**Figure 12:**
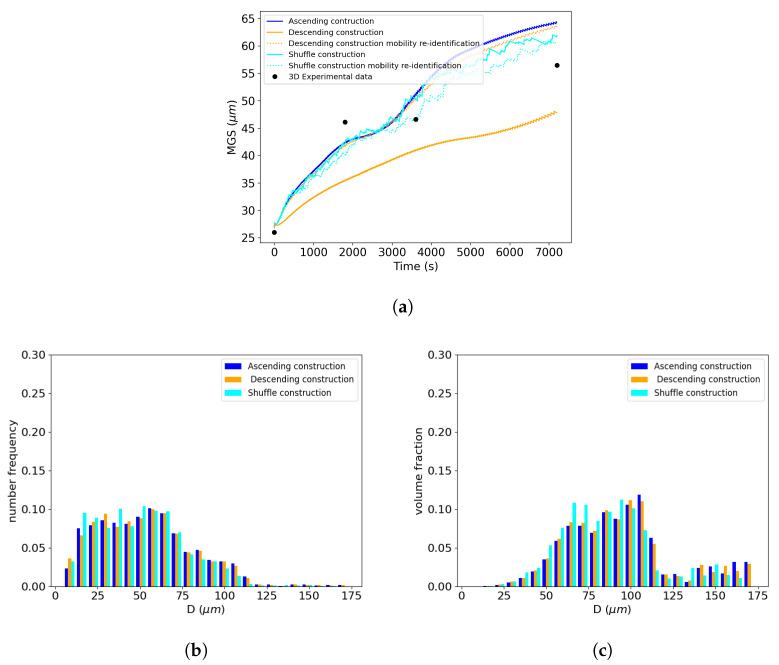
Comparison of different selection orders for the neighborhood construction using the Maire et al. model. The test case selected here is a 1h annealing time at 1100 °C. (**a**) MGS evolution with respect to time, GSD at the end of the heat treatment (*t* = 1 h) considering (**b**) the frequency of occurrence and (**c**) volume fraction.

**Figure 13:**
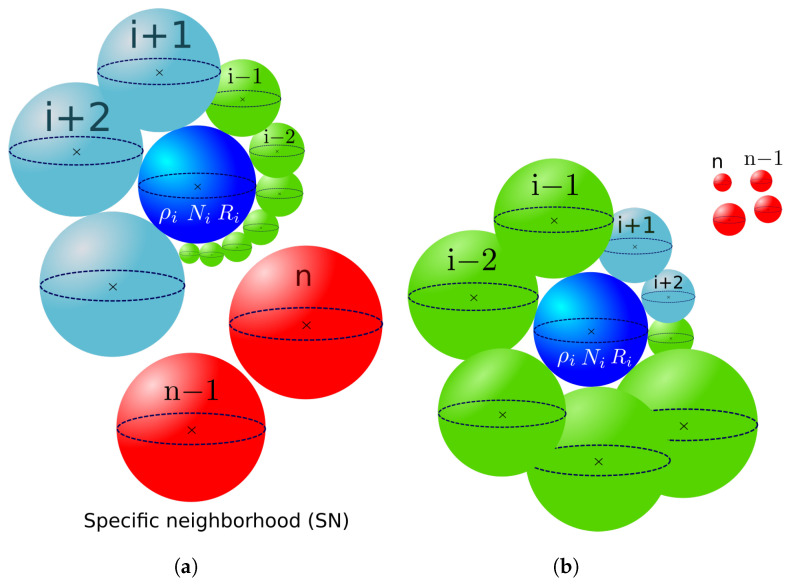
Specific neighborhood construction considering (**a**) ascending and (**b**) descending selection order in the GSD.

In Figure 12a, it can be seen that the MGS evolution was strongly affected by the selection in descending sorting order of the neighborhood construction, as indicated by the orange solid line. The shuffle order of construction had a smaller impact on the MGS kinetics compared to the descending sorting order. The reduced mobility values needed to be re-identified to achieve a good fit with the experimental data. The values for these specific selection orders of construction in the Maire et al. model are presented in Table 5 and the associated MGS evolutions are represented by the dashed lines in Figure 12a. The corresponding GSDs are presented in terms of the frequency of occurrence and volume fraction in Figure 12b,c. When the reduced mobility was re-identified, the predicted GSDs remained close to each other independently of the selected sorting order of construction. Ascending and descending construction orders tended to predict longer distribution tails than the shuffle order. However, when the mobility was not re-identified, the strong impact on MGS prediction can be explained by the difference in neighborhood construction for the same current grain class. The microstructure representations of these construction orders are schematically presented in Figure 13a,b, showing two different construction patterns. In ascending sorting order, the bigger grains of the microstructure are represented as red grain classes, i.e., those that did not participate in the neighborhood of the current class *i*. In contrast, in descending sorting order construction, the red grain classes are the smaller ones. This will modify the GB migration volume exchanges between neighboring grains during GB migration.

For the subsequent comparisons, the original ascending GSD sorting order was retained, as in this case, the reduced mobility value had an order of magnitude similar to that of the other models.

### 4.2. Comparison of Mean-Field Models Using Different Initial Microstructures

In this section, initial mono- and bimodal distributions are used to evaluate the impact of heterogeneities in the initial GSD. The monomodal test case uses the initial experimental GSD. Thermal treatment simulations are compared with experimental data. However, bimodal comparisons are made only among themselves, as no experimental data were available for this case. In both analyses, the Maire et al. model is employed with a volumetric contact probability, as detailed in Section 4.1.2, and with an initial number of grain classes of 1000, as deduced from Section 4.1.1. An ascending sorting order for the input distribution is considered, as originally used in the previous work of Maire et al. [12].

#### 4.2.1. Comparison of Mean-Field Models with a Monomodal Initial Microstructure

**Figure 14:**
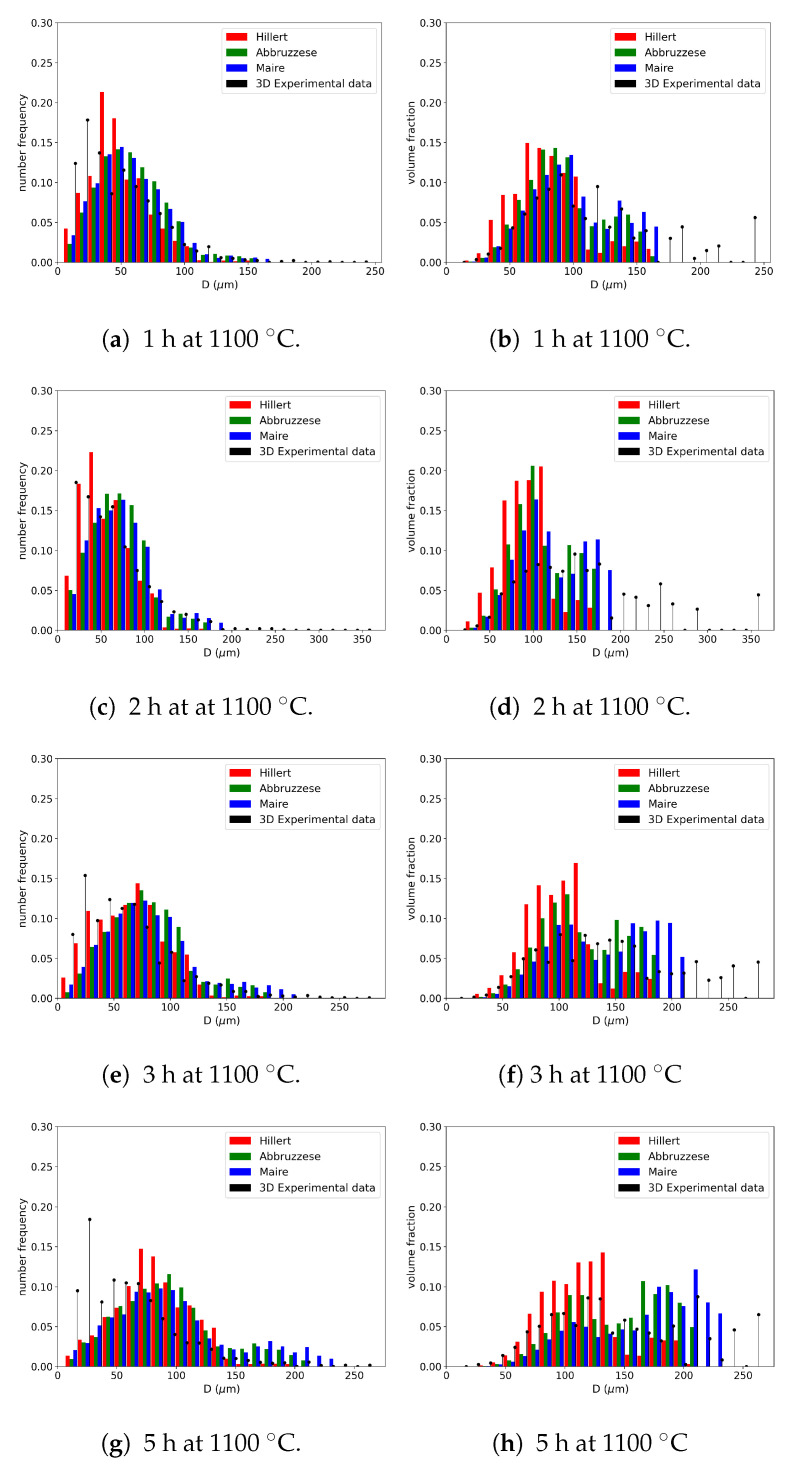
Comparisons of GSDs in terms of the (**a**,**c**,**e**,**g**) frequency of occurrence and (**b**,**d**,**f**,**h**) volume fraction for the Hillert, Abbruzzese et al., and Maire et al. models with experimental data at 1100 °C for (**a**,**b**) 1 h, (**c**,**d**) 2 h, (**e**,**f**) 3 h, and (**g**,**h**) 5 h of thermal treatment on 316L.

Figure 14a–h illustrate the mean-field GSD predictions after different annealing times at 1100 °C compared with the experimental data obtained through the EBSD. The tails of the GSDs representing larger grains were better predicted by the Maire et al. model, whether considering the frequency of occurrence or the volume fraction representation of the GSD. However, the volume fraction histograms show that none of the three models captured the entire experimental distribution tail for any of the studied annealing times. This may be due to both implementation hypotheses or to the statistical representativeness of the experimental GSD. The Hillert model seemed to better predict the frequency of occurrence histogram and tended to underestimate the volumetric predictions. However, for the Abbruzzese et al. and Maire et al. models, the initial part of the GSD, composed of small grain classes, exhibited good agreement with the experimental data in terms of the volume fraction (Figure 14a,c,e,g). On the other hand, the predictions of the frequency of occurrence showed some divergence with respect to the experimental data for small grain sizes.

One common strong hypothesis in the models was the consideration of spherical grains and their spherical evolution with capillarity. The models also made important approximations by considering the grain boundary properties as isotropic. Indeed, the grain boundary energy $\gamma_{GB}$ and its mobility $M_{GB}$ were both considered constant and identical for all grain classes used in the simulation. However, experimental microstructures have been proven [22,23] to exhibit a dispersion in the values of these properties. These hypotheses tended to smooth all microstructure heterogeneities at the beginning of or during the simulations, which may explain the GSD extremity differences. The variation in the radius exchange considered in the GB migration equation for the different microstructure descriptions also impacted the GSD prediction. Indeed, by employing an HEM, the Hillert model accounted for these variations through the choice of the MGS equation.

Experimental factors can also play a role in these differences. The statistical representativity of experimental data can be limited, and the inverse Saltykov method is not deterministic. As a result, perfect reproducibility of the data is not possible. This can explain the difficulty of the models in predicting the GSD tails. The discussed models exhibited a general tendency for the GSD evolution depending on the thermal conditions. Moreover, no special treatment was applied to consider twinning in this work, even though these special GBs were partially taken into account in the identified parameters of the models, such as $M_{GB}$.

**Figure 15:**
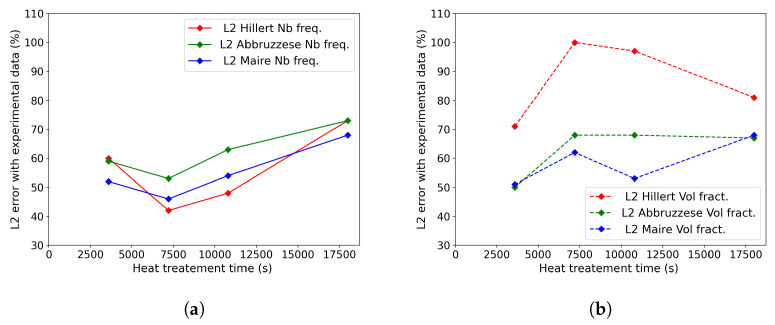
Comparison of $L^{2}\left(t\right)$ errors computed from GSDs for the three models and heat treatments from 1 h to 5 h at 1100 °C in terms of the (**a**) frequency of occurrence and (**b**) volume fraction.

For more quantified comparisons, the $L^{2}\left(t\right)$ error was computed using Equation (18), with $S_{i}$ (resp. $S_{i}^{\prime }$) corresponding to the *i*th bin grain class area of the simulated GSD at time *t* (resp. the experimental GSD at time *t*). Figure 15a,b illustrate the comparison of this criterion for the four experimented times in both representations in terms of the frequency of occurrence and volume fraction. High $L^{2}\left(t\right)$ error values can be explained by the assumptions and experimental statistical representativity detailed above. However, these results provide a good relative comparison basis between the models. As expected, the Hillert model provided a good prediction rate in terms of the frequency of occurrence. But its volumetric predictions were low compared to the other two models. The Abbruzzese et al. and Maire et al. models provided a relatively constant prediction in both representations. In order to validate a model’s prediction ability, good results in both representations are necessary. The Maire et al. model exhibited the lowest overall $L^{2}\left(t\right)$ values, making it the model with the highest accuracy compared to the other models.

In the case of the GG, overall, the simulation of an initial monomodal GSD was similarly predicted by the three models. The Maire et al. model improved the GSD predictions on larger grain sizes, but the Hillert model yielded satisfying results, considering the simplicity of its description when only the GG mechanism was at play.

#### 4.2.2. Comparison of Mean-Field Models on Bimodal Initial Microstructure

**Figure 16:**
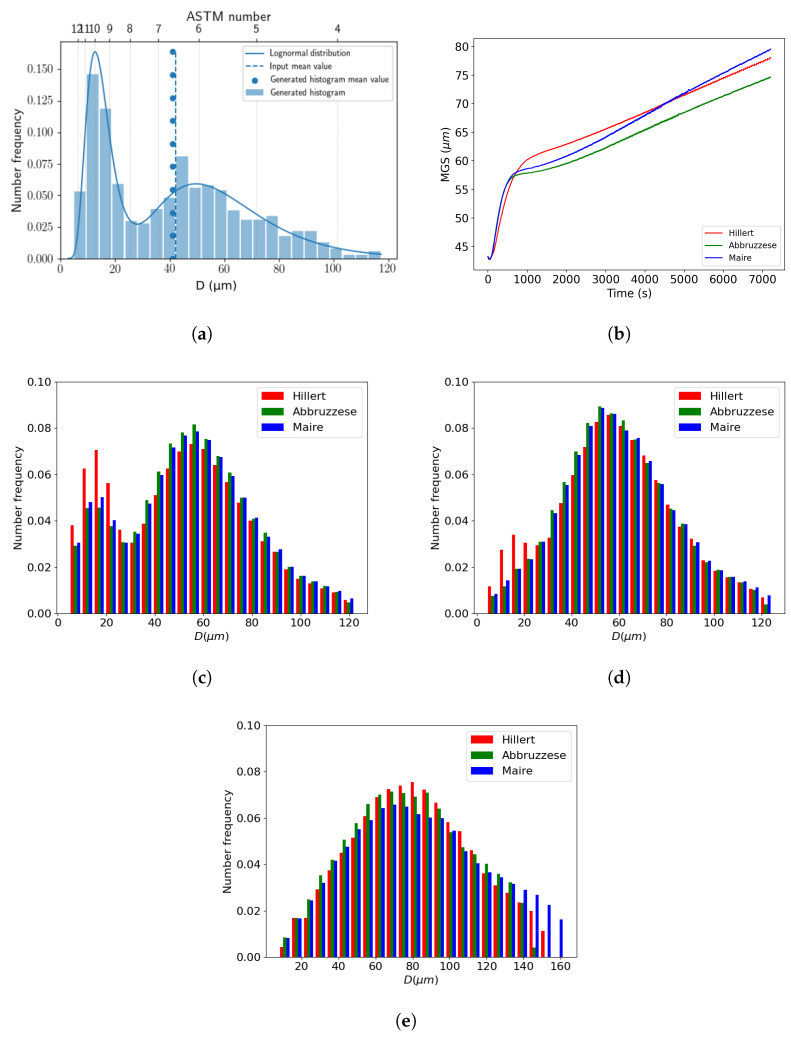
Comparison of the GG predictions at 1100 °C, starting from an initial bimodal distribution with a neighborhood construction selected in ascending order for the Maire et al. model: (**a**) Initial bimodal distribution, (**b**) MGS evolution over time, and GSDs at (**c**) 5 min, (**d**) 10 min, and (**e**) 2 h.

By developing a test case using a bimodal initial GSD, the neighborhood construction of the Maire et al. model was then tested for an initial heterogeneous GSD. An input microstructure was selected with an MGS of 42 μm and a four-ATSM grain size difference between the two selected grain populations to adhere to the bimodal definition of the ASTM standard E112 [24], as shown in Figure 16a. The previously identified reduced mobility values were used. In this case, no experimental data were available; thus, only a relative comparison between models was performed. The MGS evolution can be visually dissociated into two kinetics. The first appeared in the first 10 min, and then a more steadily increasing kinetic until the end of the annealing treatment of 2 h was observed, as shown in Figure 16b. Three associated GSDs at 5 min, 10 min, and 2 h were evident when comparing the behavior of the models, as shown in Figure 16c–e. For all cases, there were very few distinctions between the models’ predictions. Additionally, the bimodal aspect was rapidly smoothed after 5 min with the appearance of a monomodal distribution, except in the Hillert model predictions, which retained a small bimodal distribution at 5 min and 10 min. The breaking point between the two kinetics in the MGS evolution corresponded to the point where the monomodal distribution was reached after about 10 min.

##### Impact of the Selection Order of Neighborhood Construction on the Distribution Prediction

**Figure 17:**
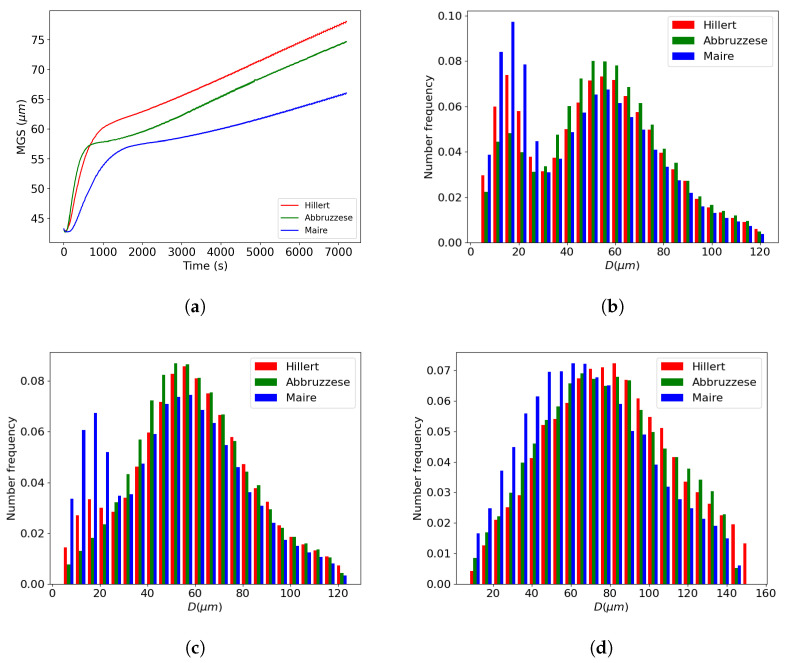
Comparison of GG predictions at 1100 °C, starting from an initial bimodal distribution with a neighborhood construction selected in descending order for the Maire et al. model: (**a**) MGS evolution over time, and GSDs at (**b**) 5 min, (**c**) 10 min, and (**d**) 2 h.

In the same way as in Section 4.1.3, observations of the use of descending selection order on the neighborhood construction were carried out. Similarly, the MGS evolutions of the Hillert and Abbruzzese et al. models, as shown in Figure 17a, remained unchanged and the kinetics of the Maire et al. model were slowed down once more. However, the associated GSDs at 5 min and 10 min effectively demonstrated the bimodality. The Maire et al. model seemed to slow down the smoothing effect on the final Gaussian distribution, as shown in Figure 17b,c. Indeed, in both graphs, the small grain size population maintained a higher frequency of occurrence in the case of the Maire et al. model compared to the other GG models. In addition to the impact of the selection order on the construction, as depicted in Figure 13, this construction technique did not allow the same neighborhood diversity for all grain classes. As shown in Figure 4, grain classes selected in the middle of the construction process benefited from a more significant number of neighbors, as part of it was built with blue grains with incomplete neighborhoods. As the end of the construction neared, fewer grain classes were available to participate in the neighborhood. Considering this point, the order of selection of neighborhood construction had an impact on the GSD prediction, even if bijectivity is taken into account. This topological information is consistent with what was observed in the GSD evolution. In the bimodal case, it appeared that ascending order construction promoted the elimination of the small grain population of the distribution with faster kinetics. Indeed, the curvature driving pressure of the GG phenomenon tended to facilitate the disappearance of smaller grains, considering the latter neighborhood construction. However, these elimination kinetics seemed to be postponed when descending order distribution was employed. In this case, small grain classes were better dispersed in the neighborhood construction and less favorable to disappearance. This led to a higher MGS in the case of the ascending order distribution of around 80 μm, whereas the descending order counterpart case barely reached 66 μm. Finally, when an analysis of the remaining grain class number at the end of the first phase at around 1000 s of heat treatment was performed, a difference of 90 grain classes in the considered system was observed. The sorting order of the grain classes using the specific neighborhood construction of the Maire et al. model had a direct impact on the evolution of the microstructure and, therefore, the GSD predictions.

## 5. Conclusions

Different GG mean-field models were investigated in this article, initially through a detailed explanation of their equations/hypotheses, and then through a comparison of heat treatment predictions for 316L steel. The neighborhood description in the Maire et al. model is based on the original work of Abbruzzese et al. [7], which describes a 2D GG model with a statistical neighborhood based on contact probabilities that involve the entire microstructure. It relies on a hybrid description by using the statistical approach of contact probabilities to define the neighbor surfaces in contact with grain classes, coupled with a deterministic number of neighbors determined by the use of a bijectivity criterion.

To optimize the accuracy of the discussed models, parameter analyses were performed to observe their impact on the GSD predictions. First, a convergence study was conducted to optimize the initial and final numbers of grain classes to ensure statistical representativity. Then, the definition of contact probability was identified as a crucial factor in the GSD description. Indeed, it impacted the distribution of the contact surface among neighboring classes. Four contact probabilities were tested, ranging from a numerical ratio not involving the grain size to a volumic fraction. This means that with a numerical contact probability, the size of the neighboring grain will only impact the grain evolution through its curvature radius in the GB migration. However, if a volumetric contact probability is considered, a more important weight will be given to large grains, emphasizing the effect of grain topology. The volumetric contact probability was selected for these reasons. In particular, this was proven to yield better GSD predictions by improving the description of the tail of the GSD when compared to experimental data, as shown in Section 4. Finally, the selected order of neighborhood construction in the GSD significantly impacted the microstructure evolution in the Maire et al. model. For this model, reduced mobility values needed to be re-identified in order to be predictive when a different selection order was adopted.

Once the optimized parameters were determined, the focus shifted to comparing the predicted GSDs with the EBSD experimental data for one to five hours of annealing. To facilitate these comparisons, it was necessary to identify the reduced mobility with respect to temperature and utilize the Saltykov method. The determined reduced mobility values ensured that the models exhibited a similar MGS evolution to the reference experimental data. On the other hand, the Saltykov method allowed for the conversion of the 2D GSD histograms to 3D discrete GSDs, providing a means for the 2D to 3D conversion of experimental EBSD data. From these comparative histograms, a good general agreement of all models with the experimental data was observed. However, the Maire et al. model yielded more satisfying results in describing the distribution tails compared to the Hillert and Abbruzzese et al. models. The $L^{2}\left(t\right)$ computation also yielded a reduced error for the Maire et al. model in the studied GG cases. When comparing implementation simplicity and the GSD response, the Hillert model generated good predictions with a simpler medium description. Another aspect of this work also involved considering other experimental data related to more heterogeneous initial grain size distributions while also taking into account the experimental data derived directly from the 3D measurements.

In this article, we have discussed the significance of the Maire et al. model solely in the context of GG. Originally designed to model the discontinuous dynamic recrystallization phenomenon [12], its strength lies in accounting for topology in the considered grain neighborhoods. It is especially powerful when dealing with different types of grains. By distinguishing RX grains from non-RX ones, the specific neighborhood construction generates better GSD predictions with respect to experimental GSDs. While this article was dedicated to validating the neighborhood construction in the case of GG, new developments aiming at improving GSD predictions during and after recrystallization are expected.

## Figures and Tables

**Table 1:** Heat treatment campaign conditions, with orange indicating the values used only for calibration, red indicating the values used for both calibration and validation, and green indicating the values used only for validation.

1000 °C	1050 °C	1100 °C
30 min	30 min	30 min
1 h	1 h	1 h
2 h	2 h	2 h
3 h	3 h	3 h
5 h	5 h	5 h

**Table 2:** Summary of post-processing details for the various isothermal treatment temperatures and holding times, including the EBSD step size (*h*), the dimensions in each direction ($L_{x}\times L_{y}$) of the analyzed areas of the sample, and the number of grains represented in the EBSD images, considering all the grains and without taking into account the twins boundaries (#G).

	*T* = 1000 °C			*T* = 1050 °C			*T* = 1100 °C		
** t **	**h (μm)**	**$L_{x}$ × $L_{y}$ (mm × mm)**	**#G**	**h (μm)**	**$L_{x}$ × $L_{y}$ (mm × mm)**	**#G**	**h (μm)**	**$L_{x}$ × $L_{y}$ (mm × mm)**	**#G**
Initial	1.49	1.1 × 0.85	980	1.49	1.1 × 0.85	980	1.49	1.1 × 0.85	980
30 min	2.5	2 × 1.4	2654	1.13	1 × 0.7	534	3.3	3.7 × 2.8	3509
1 h	2.5	2 × 1.4	2078	3	3 × 2.2	1964	3.3	3.7 × 2.8	3590
2 h	1.13	1 × 0.7	456	3	3 × 2.2	1154	3.77	3.7 × 2.8	2208
3 h	1.13	1 × 0.7	468	1.13	1 × 0.7	300	3.77	3.7 × 2.8	2263
5 h	1.13	1 × 0.7	243	1.13	1 × 0.7	133	3.77	3.7 × 2.8	2304

**Table 3:** Initial reduced mobility values ${\left(M_{GB}\gamma_{GB}\right)}_{ini}$ for the Maire et al. model in ascending order of the initial GSD for the investigated temperatures for 316L.

Temperature	1000 °C	1050 °C	1100 °C
$M_{GB}\gamma_{GB}$ (m${}^{2}$s${}^{-1}$)	2.30 × 10${}^{-15}$	1.08 × 10${}^{-13}$	1.10 × 10${}^{-13}$

**Table 4:** Identified reduced mobility values for the different models at 1100 °C for 316L.

Model	Hillert	Abbruzzese	Maire
$M_{GB}\gamma_{GB}$ (m${}^{2}$s${}^{-1}$)	1.08 × 10${}^{-13}$	1.27 × 10${}^{-13}$	1.10 × 10${}^{-13}$

**Table 5:** Identified reduced mobility values for the Maire et al. model for different selection orders at 1100 °C for 316L.

Sorting Order	Ascending	Descending	Shuffle
$M_{GB}\gamma_{GB}$ (m${}^{2}$s${}^{-1}$)	2.19 × 10${}^{-13}$	5.00 × 10${}^{-13}$	2.30 × 10${}^{-13}$

## Data Availability

The data necessary to reproduce these findings are available from the corresponding author on request.

## References

[1] Rollett A., Rohrer G.S., Humphreys J. (2017). Recrystallization and Related Annealing Phenomena.

[2] Avrami M. (1939). Kinetics of Phase Change. I. General Theory. J. Chem. Phys..

[3] Johnson W., Mehl R. (1939). Reaction kinetics in processes of nucleation and growth. Trans. Am. Inst. Min. Engin..

[4] Bernacki M., Chastel Y., Coupez T., Logé R. (2008). Level set framework for the numerical modelling of primary recrystallization in polycrystalline materials. Scr. Mater..

[5] Hallberg H. (2011). Approaches to Modeling of Recrystallization. Metals.

[6] Hillert M. (1965). On the theory of normal and abnormal grain growth. Acta Metall..

[7] Abbruzzese G., Heckelmann I., Lücke K. (1992). Statistical theory of two-dimensional grain growth—I. The topological foundation. Acta Metall. Mater..

[8] Montheillet F., Lurdos O., Damamme G. (2009). A grain scale approach for modeling steady-state discontinuous dynamic recrystallization. Acta Mater..

[9] Cram D., Zurob H., Brechet Y., Hutchinson C. (2009). Modelling discontinuous dynamic recrystallization using a physically based model for nucleation. Acta Mater..

[10] Bernard P., Bag S., Huang K., Logé R. (2011). A two-site mean field model of discontinuous dynamic recrystallization. Mater. Sci. Eng. A.

[11] Favre J., Fabrègue D., Piot D., Tang N., Koizumi Y., Maire E., Chiba A. (2013). Modeling Grain Boundary Motion and Dynamic Recrystallization in Pure Metals. Metall. Mater. Trans. A.

[12] Maire L., Fausty J., Bernacki M., Bozzolo N., Micheli P.D., Moussa C. (2018). A new topological approach for the mean field modeling of dynamic recrystallization. Mater. Des..

[13] Burke J., Turnbull D. (1952). Recrystallization and grain growth. Prog. Metal Phys..

[14] Lücke K., Heckelmann I., Abbruzzese G. (1992). Statistical theory of two-dimensional grain growth—II. Kinetics of grain growth. Acta Metall. Mater..

[15] Beltran O., Huang K., Logé R. (2015). A mean field model of dynamic and post-dynamic recrystallization predicting kinetics, grain size and flow stress. Comput. Mater. Sci..

[16] Flipon B., Bozzolo N., Bernacki M. (2022). A simplified intragranular description of dislocation density heterogeneities to improve dynamically recrystallized grain size predictions. Materialia.

[17] Bachmann F., Hielscher R., Schaeben H. (2010). Texture Analysis with MTEX—Free and Open Source Software Toolbox. Solid State Phenom..

[18] Saltykov S. (1958). Stereometric Metallography.

[19] Tucker J.C., Chan L.H., Rohrer G.S., Groeber M.A., Rollett A.D. (2012). Comparison of grain size distributions in a Ni-based superalloy in three and two dimensions using the Saltykov method. Scr. Mater..

[20] Lopez-Sanchez M., Llana-Fúnez S. (2016). An extension of the Saltykov method to quantify 3D grain size distributions in mylonites. J. Struct. Geol..

[21] Di Schino A., Kenny J.M., Salvatori I., Abbruzzese G. (2001). Modelling primary recrystallization and grain growth in a low nickel austenitic stainless steel. J. Mater. Sci..

[22] Rohrer G.S. (2010). “Introduction to Grains, Phases, and Interfaces—An Interpretation of Microstructure,” *Trans. AIME*, 1948, vol. 175, pp. 15–51, by CS Smith. Metall. Mater. Trans. A.

[23] Kohara S., Parthasarathi M.N., Beck P.A. (1958). Anisotropy of Boundary Mobility. J. Appl. Phys..

[24] (2004). Standard Test Methods for Determining Average Grain Size.

